# Genetic variation of *Glucose Transporter-1 *(*GLUT1*) and albuminuria in 10,278 European Americans and African Americans: a case-control study in the Atherosclerosis Risk in Communities (ARIC) Study

**DOI:** 10.1186/1471-2350-12-16

**Published:** 2011-01-19

**Authors:** Charles C Hsu, Wenhong L Kao, Michael W Steffes, Tejal Gambir, Frederick L Brancati, Charles W Heilig, Alan R Shuldiner, Eric A Boerwinkle, Josef Coresh

**Affiliations:** 1 Departments of Medicine and Epidemiology, Johns Hopkins Medical Institutions, Baltimore, Maryland, USA; 2 Department of Laboratory Medicine and Pathology, University of Minnesota, Minneapolis, Minnesota, USA; 3 Department of Medicine, University of Maryland School of Medicine, Baltimore, Maryland, USA; 4 Human Genetics Center, University of Texas-Houston Health Science Center, Houston, Texas, USA; 5 Department of Medicine, University of Chicago, Chicago, Illinois, USA; 6 Department of Radiation Oncology, University of California, San Francisco, California, USA

## Abstract

**Background:**

Evidence suggests glucose transporter-1(*GLUT1*) genetic variation affects diabetic nephropathy and albuminuria. Our aim was to evaluate associations with albuminuria of six *GLUT1 *single nucleotide polymorphisms(SNPs), particularly *XbaI *and the previously associated *Enhancer-2(Enh2*) SNP.

**Methods:**

A two-stage case-control study was nested in a prospective cohort study of 2156 African Americans and 8122 European Americans with urinary albumin-to-creatinine ratio(ACR). Cases comprised albuminuria(N = 825; ≥ 30 μg/mg) and macroalbuminuria(N = 173; ≥ 300 μg/mg). ACR < 30 μg/mg classified controls(n = 9453). Logistic regression and odds ratios(OR) assessed associations. The evaluation phase(stage 1, n = 2938) tested associations of albuminuria(n = 305) with six *GLUT1 *SNPs: rs841839, rs3768043, rs2297977, *Enh2*(rs841847) *Xba*I(rs841853), and rs841858. *Enh2 *was examined separately in the replication phase(stage 2, n = 7340) and the total combined sample (n = 10,278), with all analyses stratified by race and type 2 diabetes.

**Results:**

In European Americans, after adjusting for diabetes and other *GLUT1 *SNPs in stage 1, *Enh2 *risk genotype(TT) was more common in albuminuric cases(OR = 3.37, P = 0.090) whereas *XbaI *(OR = 0.94, p = 0.931) and remaining SNPs were not. In stage 1, the *Enh2 *association with albuminuria was significant among diabetic European Americans(OR = 2.36, P = 0.025). In African Americans, *Enh2 *homozygosity was rare(0.3%); *XbaI *was common(18.0% AA) and not associated with albuminuria. In stage 2(n = 7,340), *Enh2 *risk genotype had increased but non-significant OR among diabetic European Americans(OR = 1.66, P = 0.192) and not non-diabetics(OR = 0.99, p = 0.953), not replicating stage 1. Combining stages 1 and 2, *Enh2 *was associated with albuminuria(OR 2.14 [1.20-3.80], P = 0.009) and macroalbuminuria(OR 2.69, [1.02-7.09], P = 0.045) in diabetic European Americans. The *Enh2 *association with macroalbuminuria among non-diabetic European Americans with fasting insulin(OR = 1.84, P = 0.210) was stronger at the highest insulin quartile(OR = 4.08, P = 0.040).

**Conclusions:**

As demonstrated with type 1 diabetic nephropathy, the *GLUT1 Enh2 *risk genotype, instead of *Xba*I, may be associated with type 2 diabetic albuminuria among European Americans, though an association is not conclusive. The association among diabetic European Americans found in stage 1 was not replicated in stage 2; however, this risk association was evident after combining all diabetic European Americans from both stages. Additionally, our results suggest this association may extend to non-diabetics with high insulin concentrations. Rarity of the *Enh2 *risk genotype among African Americans precludes any definitive conclusions, although data suggest a risk-enhancing role.

## Background

Glucose transporter 1(GLUT1) is the major facilitative glucose transporter in glomerular mesangial cells[1]. Studies show glucose transport as the rate limiting step in mesangial expansion, and alterations of GLUT1 activity may stimulate extracellular matrix(ECM) production [2] even under normoglycemic conditions [3]. Additionally, experimental evidence suggests GLUT1 may be associated with hypertensive glomerulopathy[4].

**Figure 1:**
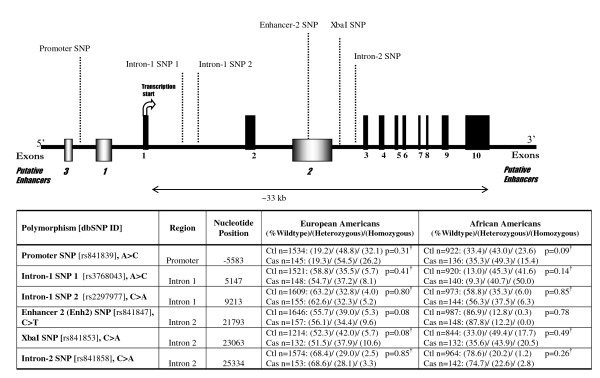
***GLUT1 *is located on chromosome 1p34.2**. The genomic structure of *GLUT1 *is illustrated with its 10 exons and three putative enhancers[5]. Genotyped polymorphisms located in intron 2 include the intron-2 SNP (rs841858), and the *Enh2*(rs841847) and *XbaI *(rs841853) SNPs which have been previously associated with diabetic nephropathy[5-7]. In the distal promoter region, the Promoter SNP (rs841839) was genotyped (located between putative enhancers 3 and 1). We also included two SNPs in intron 1, intron-1 SNP(rs3768043) and intron-2 SNP(rs2297977). No SNP was associated with albuminuria among all African Americans or among all European Americans, including XbaI (p = 0.08) and Enh2 (p = 0.08). None of the tagging SNPs were able to reach a Bonferroni adjusted level of statistical significance. ^†^Bonferroni corrected level of statistical significance p = 0.01.

However, results of case-control studies of GLUT1 sequenece variations(the *Xba*I polymorphism, a C-to-A transversion in intron 2: see figure 1) and diabetic nephropathy have been inconsistent[5-11] with significant heterogeneity between studies[12]. Most studies demonstrated risk transmitted in a recessive fashion[5,6,8,11,13]. Few studies examined diabetic nephropathy in relation to *GLUT1 *SNPs other than XbaI[5,11,13]. A study of those with type 1 diabetes examined six *GLUT1 *single nucleotide polymorphisms(SNPs) and found homozygosity for the *Xba*I A allele was associated with diabetic nephropathy[5]. Additionally, putative human enhancer elements were identified [5], including the insulin-responsive enhancer-2(see figure 1), and homozygosity for the minor allele(C-to-T) of the enhancer-2 SNP1(*Enh2 *SNP) was also associated with type 1 diabetic nephropathy[5]. Though Enh2 SNP has strong linkage disequilibrium with XbaI SNP, more evidence suggests Enh2 SNP may be causally related to diabetic nephropathy[5]. More recently, a study extended the significant association of the *Enh2 *SNP risk genotype to those with type 2 diabetic nephropathy; however, a statistically significant association between *Xba*I genotypes and type 2 diabetic nephropathy was not demonstrated [11].

There has been no investigation among non-diabetics or African Americans of albuminuria and *GLUT1 *genetic variation, especially for the *Enh2 *SNP. Additionally, the *Enh2 *SNP is not readily available on most candidate gene chips, with available proxy SNPs having low r^2^. We conducted a case-control study nested within a large biracial community-based middle-aged cohort to determine the risk of albuminuria associated with six GLUT1 SNPs by race and by type 2 diabetes status. We performed a two-stage analysis in our population(n = 10,278): 1) an initial evaluation of all six candidate GLUT1 SNPs among stage 1 participants(n = 2938), and 2) a subsequent phase (stage 2, n = 7340) to assess the likeliest stage 1 candidate SNP. Because enhancer-2 is a putative insulin-responsive element[5], we hypothesized insulin may interact with the Enh2 SNP to affect risk of albuminuria.

## Methods

### Study Design and Population

The study population consisted of African Americans and European Americans with and without type 2 diabetes in the Atheroslcerosis Risk in Communities (ARIC) Study, aged 45 to 64 years at baseline in 1987 through 1989, from 4 US communities: Forsyth County, NC; Jackson, MS; suburbs of Minneapolis, MN, and Washington Co., MD. Participants underwent four standardized examinations in field center clinics, scheduled approximately every three years[14] with approval of the institutional review boards.

Of the 11,625 African American and European American participants who attended visit four, urinary albumin and creatinine measurements were available for 11,447 participants. All measurements were from ARIC visit 4, as urine samples were only collected at this visit. We excluded those missing visit 4 serum creatinine (n = 182), information on diabetes, hypertension, systolic blood pressure (SBP), diastolic blood pressure (DBP), or body mass index (BMI) (n = 125), and those without available DNA (n = 69). For stage 1, we genotyped six *GLUT1 *SNPs (including Enh2 and XbaI) in a sample of the above participants (sample 1, n = 3,204), enriched for African Americans and people with type 2 diabetes, a convenience sample previously used for the study of genetic risk factors in cardiovascular disease and type 2 diabetes. Sample 1 was an otherwise unselected portion of the total population. We genotyped only the most likely candidate SNP (Enh2) from stage 1 in the remaining participants who met our inclusion criteria from ARIC visit 4, individuals who comprised stage 2 (sample 2, n = 7,867). Missing genotypes were excluded from stages 1(n = 266) and 2(n = 527). With exclusion criteria, of the total study population (n = 10,278), sample 1(n = 2938) was 28.6% and sample 2(n = 7340) was 71.4%. Participants from samples 1 and 2 were mutually exclusive.

Of those who attended visit 4, the 1,347 excluded individuals were older (63.1 vs. 62.8 years of age), more likely to be African American (37.7% vs. 21.0%), type 2 diabetic (22.5% vs. 16.2%), hypertensive (57.2% vs. 46.7%), and male (46.5% vs. 43.8%) compared to included participants.

### Assessment of Clinical Characteristics

Clinical charcteristics were assessed during visit 4. A standardized interview, clinical examination, and laboratory investigation collected demographic, anthropometric, and cardiovascular risk factor data for participants [15]. Fasting blood sample collection and processing is described elsewhere [15]. Two standardized blood pressure measurements were performed by trained technicians, and their average was used. Hypertension was defined as a systolic blood pressure (SBP) ≥140 mmHg, a diastolic blood pressure (DBP) ≥90 mmHg, or the self-reported use of antihypertensive medication during the previous 2 weeks. Diabetes mellitus was defined as fasting glucose ≥ 126 mg/dL, nonfasting glucose ≥200 mg/dL, or self-reported history or treatment of type 2 diabetes. Visit 4 glomerular filtration rate (GFR) was estimated from calibrated serum creatinine using the simplified equation developed using data from the Modification of Diet in Renal Disease (MDRD) Study[16,17] as follows:

GFR mL/min/1.73m^2 ^= 186.3 * (SCr)^-1.154 ^* (age)^-0.203 ^* (0.742 if female) * (1.21 if African American)

### Ascertainment of Albuminuria

An untimed urine sample was collected during visit 4. Aliquots were frozen within 12 hours and stored at -70°C. Albumin and creatinine concentrations were measured in the University of Minnesota Physicians Outreach Laboratories, Minneapolis, Minnesota, with albumin by a nephelometric method on the Dade Behring BN100 (assay sensitivity, 2.0 mg/L), and creatinine using the Jaffe method on a Beckman CX3 to determine albumin-to-creatinine ratios (ACR; ug/mg) for participants. Blinded samples (n = 516) analyzed for quality assurance showed a correlation coefficient^® ^of the log_e_-transformed ACR as r = 0.95. ACR was used to determine levels of albuminuria according to American Diabetes Association [18] and National Kidney Foundation [19] recommendations: normoalbuminuria (ACR < 30 μg/mg), microalbuminuria (ACR 30-299 μg/mg), and macroalbuminuria (ACR ≥ 300 μg/mg). Normoalbuminuric participants were classified as controls. Albuminuria cases had either microalbuminuria or macroalbuminuria. Of the total study, there were 825 cases of albuminuria, 37.0%(n = 305) in sample 1 and 63.0%(n = 520) in sample 2. For sensitivity analysis, microalbuminuria and macroalbuminuria cases were examined separately, compared to controls.

### GLUT1 Candidate SNP Selection and Genotyping

*GLUT1 *consists of 10 exons including three recently described putative human enhancers (see Figure 1)[5] with murine and rat homology[20,21]. The XbaI SNP[5-7] and the *Enh2 *SNP[5,11] were chosen as candidates for genotyping based on previous studies. Using the human *GLUT1 *genomic sequence(GenBank accession no. NT_032977) and the public SNP database dbSNP build 120, it was determined the XbaI SNP corresponds to rs841853 and the Enh2 SNP corresponds to rs841847. The dbSNP rs841839 C > A transversion(denoted Promoter SNP) was chosen for genotyping based on its proximity to both enhancers-3 and -1. At the time of genotyping, the Hap Map Phase II data were not available; therefore, 3 additional SNPs were chosen with an average distance between SNPs of 4.2 kb to provide coverage of the gene (76.8%). Three additional SNPs selected were: intron-1 SNP1(rs3768043), intron-1 SNP2(rs2297977), and intron-2 SNP(rs841858).

For the evaluation phase, genotypes in sample 1 were assessed by PCR amplification of genomic DNA and completed for rs841847(Enh2 SNP) using Pyrosequencing technology (Pyrosequencing, Uppsala, Sweden) as previously described [22]. For the SNPs rs841853 (XbaI), rs841839, rs3768043, rs2297977, and rs841858, genotypes were completed using Orchid SNPstream UHT genotyping system (Orchid Bioscience) as previously described[23]. For sample 1, the error rate on the basis of blind replicates (n = 444) for the Orchid SNPstream UHT genotyping system was 0.2%. For the replication phase, genotypes for rs841847 in sample 2 were assessed and completed using Pyrosequencing technology.

### Statistical Analysis

For stage 1(n = 2938), genotype frequencies of all SNPs were tested for consistency with Hardy-Weinberg expectations by the χ^2 ^test within race strata among controls consisting of non-diabetic individuals without albuminuria. Because allele frequencies differed by race, analyses were race-stratified. Tests of differences in clinical characteristics by case status included t-tests, ANOVA, and χ^2 ^tests. Logistic regression models calculated the odds ratio (OR) and 95% confidence interval (95% CI) of albuminuria for each SNP, using either genotypic or recessive models for inheritance. Our genotypic associations of XbaI and Enh2 in European Americans suggest recessive modes of action, similar to previous findings [5,6]. To differentiate the effects of the likely candidate from remaining GLUT1 SNPs, multivariate logistic regression was performed, adjusting for type 2 diabetes, XbaI, Enh2, rs841839, rs841858, rs2297977, and rs3768043 genotypes.

There was strong prior evidence for Enh2 (rs841847) as a functional candidate polymorphism, both in human and laboratory studies [5,11,24-29]. Based on this *a priori *determination of the status of Enh2 (rs841847) as a functional candidate SNP, we believed it was appropriate to keep the alpha for statistical significance of p < 0.05 for analyses focusing on Enh2 (rs841847) with further correction. However, given that the other 5 SNPs (rs841853, rs841839, rs3768043, rs2297977, rs841858) examined were largely meant to be tagging SNPs without any prior evidence to suggest function, we felt it necessary to correct for multiple testing errors by applying a Bonferroni correction[30], with the corrected alpha for statistical significance at the 0.05 level determined by dividing the significance level by the number of tagging SNPs, with adjusted p-value < 0.01.

Using the Bayesian method as implemented in PHASE v2.1[31,32], haplotypes were inferred for all individuals with genotype data for at least five of the six SNPs (rs841847, rs841853, rs841839, rs3768043, rs2297977, rs841858) separately for African Americans (n = 1,036) and European Americans (n = 1638). Phase-formatted data were run as race-specific files which combined case and control subjects, a more conservative estimation of haplotype frequency than separate case and control sample analyses. Pairwise SNP linkage disequilibrium (LD) was measured with D' and r^2^[33] in HaploView(Cambridge, MA). Categorized by major haplotypes (> 5% frequency), haplotype frequency was compared between case and control groups with χ^2 ^test. For haplotype analyses, a Bonferroni correction was also applied by dividing the significance level (0.05) by the number of major haplotypes (n = 4 for European Americans, n = 8 for African Americans) for haplotype-based association analysis (Bonferroni corrected level of statistical significance for European Americans p < 0.0125, for African Americans p < 0.00625). Associations with albuminuria for each diplotype (of major haplotypes) were examined separately using logistic regression. For diplotype analyses for European Americans, a Bonferroni correction was also applied by dividing the significance level by the number of diplotypes (n = 10) comprised of the major haplotypes, with adjusted level of statistical significance p < 0.005.

In stage 2 (n = 7340) the Enh2 SNP was genotyped. Associations of albuminuria/microabluminuria/macroalbuminuria and the Enh2 risk genotype were assessed using multivariate logistic regression models for the total study population, stratified by race and type 2 diabetes. Covariates included age, gender, systolic blood pressure, diastolic blood pressure, hypertension medication use, BMI, estimated GFR, and fasting glucose. Additionally, among non-diabetics, we examined interactions of albuminuria/macroalbuminuria for the Enh2 risk genotype with quartiles of fasting insulin, based on the Enh2 SNP location in the putative insulin-responsive enhancer 2[5]. Multivariate linear regression was used to assess associations between the Enh2 risk genotype with log-transformed ACR μg/mg (data log-transformed due to the skewed distribution) and also serum creatinine among European Americans, stratified by type 2 diabetes; these analyses were not performed among African Americans due to the rarity of the Enh2 risk genotype in that population.

## Results

### *GLUT1 *SNPs

Six SNPs (average spacing 4.2 kb) of the *GLUT1 *genomic region (Promoter SNP, Intron-1 SNPs 1 and 2, Enhancer 2 SNP, XbaI SNP, and Intron 2 SNP) were genotyped in an unrelated sample of 1803 European Americans and 1135 African Americans in stage 1 (Figure 1). In European Americans, all SNPs were in Hardy-Weinberg Equilibrium (HWE). Among African Americans, the Promoter SNP was not in HWE (P < 0.05). *XbaI *minor allele(A) frequency in African Americans was higher than in European Americans (frequency = 0.42 versus 0.27). In European Americans, the *XbaI *A allele and the *Enh2 *T allele (frequency = 0.25) were comparable to previously published estimates[5,6,8,9,11]. However, minor allele of *Enh2*(T) was low in African Americans (frequency = 0.07).

The five SNPs (Intron-1 SNPs 1 and 2, Enh2 SNP, XbaI SNP, and Intron-2 SNP) downstream of Promoter SNP were in strong LD with each other (range of D' = 0.95 to 1.00 in European Americans and 0.96 to 1.00 in African Americans)[34]. The promoter SNP was separated from Intron-1 SNP1 by ~10 kb and was in greater LD among European Americans (D' = 0.69) than African Americans (D' = 0.09). Among both African Americans and European Americans, Enh2 and XbaI SNPs were in strong LD (D' = 0.96); however, the *r*^*2*^was much lower in African Americans compared to European Americans (0.09 vs. 0.70) because frequencies of Enh2 and XbaI significantly differed in African Americans.

### Clinical Characteristics

In both race groups in stage 1, the case subjects had worse risk factor profiles than controls (Table 1). Risk factor profiles were also worse for case subjects among participants from stage 2 (n = 7340).

**Table 1:** Baseline Characteristics. Clinical characteristics of study subjects by Race and Albuminuric Status

	Stage 1				Stage 2			
	**European Americans (n = 1803)**				**European Americans (n = 6319)**			
**Characteristic**	**Control Subjects**		**Albuminuria Cases**		**Control Subjects**		**Albuminuria Cases**	

**N**	1646		157		5945		374	
**Age, mean(SD), y**	63.2	(5.6)	64.9	(5.6)***	62.8	(5.6)	65.2	(5.6)***
**Male, No. (%)**	802	(48.7)	92	(58.6)*	2655	(44.7)	199	(53.2)**
**Hypertension, No. (%)**	731	(44.4)	111	(70.7)***	2266	(38.1)	269	(71.9)***
**Type 2 Diabetes, No. (%)**	480	(29.2)	84	(53.5)***	428	(7.2)	103	(27.5)***
**Body mass index, kg/m**^**2**^	29.0	(5.5)	30.0	(6.3)*	28.0	(5.0)	28.8	(6.0)**
**SBP (mmHg)**	126.8	(17.4)	135.7	(20.4)***	124.4	(17.8)	138.3	(23.2)***
**DBP (mmgHg)**	69.7	(10.0)	70.0	(11.1)	69.5	(9.6)	72.5	(12.1)***
**Fasting Glucose (mg/dL)**^**§**^	115.4	(34.9)	134.4	(52.2)***	102.1	(21.2)	116.8	(43.4)***
**GFR, ml/min per 1.73 m2**	81.7	(16.9)	75.0	(25.7)***	80.7	(16.1)	76.1	(22.3)***
**ACR, μg/mg, median (25%ile, 75%ile)**	3.7	(1.8,6.7)	102.6	(47.4,275.0)***	3.6	(2.0,6.5)	74.8	(44.4,166.7)***

								
	**African Americans (n = 1135)**				**African Americans (n = 1021)**			
**Characteristic**	**Control Subjects**		**Albuminuria Cases**		**Control Subjects**		**Albuminuria Cases**	

**N**	987		148		875		146	
**Age, mean(SD), y**	61.3	(5.5)	63.0	(5.8)^†††^	61.8	(5.7)	62.5	(5.7)
**Male, No. (%)**	336	(34.0)	44	(29.7)	324	(37.0)	54	(37.0)
**Hypertension, No. (%)**	615	(62.3)	127	(85.8)^†††^	558	(63.8)	127	(87.0) ^†††^
**Type 2 Diabetes, No. (%)**	217	(22.0)	80	(54.1)^†††^	196	(22.4)	80	(54.8) ^†††^
**Body mass index, kg/m**^**2**^	30.7	(6.5)	30.8	(6.6)	30.3	(5.9)	31.4	(6.6) ^†^
**SBP (mmHg)**	131.2	(18.4)	144.4	(23.2)^†††^	132.0	(18.9)	144.8	(22.5) ^†††^
**DBP (mmgHg)**	75.0	(10.0)	78.2	(13.0)^†††^	75.7	(10.1)	78.5	(11.9) ^††^
**Fasting Glucose (mg/dL) **^**§**^	114.3	(39.6)	136.4	(62.2)^†††^	112.7	(38.5)	137.9	(62.8) ^†††^
**GFR, ml/min per 1.73 m2**	91.5	(19.0)	83.0	(29.8) ^†††^	90.9	(21.0)	80.7	(29.3) ^†††^
**ACR, μg/mg, median (25%ile, 75%ile)**	1.9	(0.7,4.7)	95.9	(47.2,363.2)^†††^	2.2	(0.8,5.2)	81.7	(44.7,249.7) ^†††^

Data are means (SD), number (%), or for ACR median (25%ile, 75%ile). European Americans: * P < 0.05; **P < 0.01; ***P < 0.001.

African Americans: †P < 0.05; ††P < 0.01; †††P < 0.001.

^§^Individuals with fasting glucose measured. Evaluation set (European Americans n = 1582 controls and n = 150 cases; African Americans n = 922 controls and n = 113 cases). Valdiation set (European Americans n = 5822 controls and n = 356 cases; African Americans n = 787 controls and n = 113 cases).

### Stage 1: Evaluation Phase of Six GLUT1 SNP Genotypes by Albuminuria Case Status

#### European Americans

Among European Americans in stage 1, *GLUT1 *SNP genotype distributions were not significantly different by case status, though the Enh2 SNP approached statistical significance (P = 0.08, figure 1). XbaI and other tagging SNPs did not approach the Bonferroni corrected level of statistical significance of p < 0.01. For *Enh2 *T allele, heterozygotes had an OR = 0.88, while homozygotes had OR = 1.77 (95% CI: 0.98 - 3.20). Similarly for the *XbaI *A allele, heterozygotes had OR 0.92 while homozygotes had OR 1.89 (95% CI: 1.01 - 3.55). Our genotypic associations of XbaI and Enh2 in European Americans suggest a recessive model, similar to previous findings [5,6,11].

Albuminuria was associated with the Enh2 TT risk genotype with OR 2.36 (95% CI: 1.09 - 5.09, P = 0.025) compared to those without the risk genotype (CC or CT) among those with type 2 diabetes. This association was not present among non-diabetics (OR 1.31, P = 0.574, Table 2). Similarly, the *XbaI *risk genotype (AA) had a larger association with albuminuria among those with type 2 diabetes rather than non-diabetics (OR 2.23 [P = 0.064] and 1.75 [P = 0.212], respectively), though associations were not statistically significant for *XbaI *and did not approach the Bonferroni corrected level of statistical significance (p < 0.01) (Table 2). Among European Americans stratified by diabetes, besides *Enh2 *no other *GLUT1 *polymorphisms were associated with albuminuria (results not shown). In all European Americans, multivariate analysis adjusting for diabetes simultaneously examined the association with albuminuria of all six GLUT1 SNPs, and the *Enh2 *risk genotype had an increased OR with albuminuria (OR = 3.37, 95% CI: 0.83 - 13.78, p = 0.090) whereas XbaI did not (OR = 0.94, 95% CI: 0.26-3.42, p = 0.931). For the four remaining SNPs, genotypic ORs ranged from 0.72 to 1.85 (all p-values > 0.17) with none of the SNPs approaching the Bonferroni adjusted statistical significance level of p < 0.01.

**Table 2:** Stage 1*GLUT1 *Enh2 and XbaI Risk genotype distributions among case and control subjects and odds ratio of albuminuria, by diabetes and race

	European Americans									
	**Type 2 Diabetes**					**No Type 2 Diabetes**				

**SNP**	**Controls**	**Cases**	**P-value**	**OR (95% CI)**		**Controls**	**Cases**	**P-value**	**OR (95% CI)**	

***Enh2 *SNP (C > T)**	480	84				1166	73			

**CC or CT**	(94.6)	(88.1)	0.025	1.00		(94.7)	(93.1)	0.574	1.00	
**TT**	(5.4)	(11.9)		2.36	(1.09 - 5.09)*	(5.3)	(6.9)		1.31	(0.51 - 3.36)

										

***XbaI *SNP (C > A) **^**†**^	348	70				866	62			

**CC or CA**	(94.5)	(88.6)	0.064	1.00		(94.2)	(90.3)	0.212	1.00	
**AA**	(5.5)	(11.4)		2.23	(0.94 - 5.33)	(5.8)	(9.7)		1.75	(0.72 - 4.25)

										

	**African Americans**									

	**Type 2 Diabetes**					**No Type 2 Diabetes**				

**SNP**	**Controls**	**Cases**	**P-value**	**OR (95% CI)**		**Controls**	**Cases**	**P-value**	**OR (95% CI)**	

***Enh2 *SNP (C > T)**	217	80				770	68			

**CC or CT**	(99.5)	(100.0)	0.543	1.00		(99.7)	(100.0)	0.674	1.00	
**TT**	(0.5)	(0.0)		N/A		(0.3)	(0.0)		N/A	

										

***XbaI *SNP (C > A) **^**†**^	190	73				654	59			

**CC or CA**	(81.6)	(80.8)	0.888	1.00		(82.6)	(78.0)	0.376	1.00	
**AA**	(18.4)	(19.2)		1.05	(0.53 - 2.09)	(17.4)	(22.0)		1.34	(0.70 - 2.56)

*P < 0.05.

^†^Bonferroni correction requires p < 0.01 to reach statistical significance

Similarly, detailed haplotype and diplotype analyses supported a potential role of the Enh2 TT genotype in raising the risk of albuminuria in a recessive fashion (Table 3). When the ten diplotypes associated with the major haplotypes A, B, C, and D (table 3) were examined among European Americans, only the BD diplotype was associated with albuminuria (OR 3.03, 95% CI 1.36 - 6.79, p = 0.007, compared to individuals without the BD diplotype). However, this approached but did not reach a Bonferroni corrected level of statistical significance of p < 0.005. Additionally, the diplotypes consisting of the B or D "risk" haplotypes were rare among European Americans, with BB, BD, and DD diplotypes accounting for 2.7%, 2.2%, and 0.5% of the 1663 European Americans with available diplotypes analyzed. Overall, haplotype diplotypes homozygous for the *Enh2 *T allele had an increased risk of albuminuria compared to all others combined (P = 0.014), though it did not reach the Bonferroni corrected level of statistical significance of p < 0.005.

**Table 3:** Major Haplotype Distribution in Case and Control Subjects by Race in stage 1^†‡^

European Americans	Haplotype	Promoter SNP	Intron-1 SNP1	Intron-1 SNP2	*Enh2 *SNP	*Xba*I SNP	Intron-2 SNP	Controls	Albuminuria Cases	P-value
**N = 3326 Haplotypes**	**A**	C	A	C	C	C	C	(43.4)	(39.5)	0.773
	**B**	A	A	C	T	A	C	(17.2)	(18.9)	
	**C**	A	C	A	C	C	A	(14.1)	(14.5)	
	**D**	C	A	C	T	A	C	(6.2)	(6.8)	
	**Others**	*	*	*	*	*	*	(19.1)	(20.3)	
**African Americans**										
**N = 2156 Haplotypes**	**A**	C	A	C	C	C	C	(13.7)	(10.7)	0.877
	**B**	A	A	C	T	A	C	(5.4)	(5.0)	
	**C**	A	C	A	C	C	A	(7.7)	(9.6)	
	**E**	C	C	C	C	C	C	(14.0)	(13.9)	
	**F**	C	C	C	C	A	C	(11.0)	(12.1)	
	**G**	A	C	C	C	A	C	(12.7)	(11.4)	
	**H**	A	C	A	C	C	C	(11.9)	(12.1)	
	**I**	A	A	C	C	A	C	(6.8)	(7.5)	
	**Others**	*	*	*	*	*	*	(16.8)	(17.5)	

‡ Major haplotypes have a frequency ≥ 5%. Minor alleles are shaded in gray. Bonferroni corrected level of statistical significance for European Americans p < 0.0125 and for African Americans p < 0.00625.

^† ^Among European Americans, the ten diplotypes associated with the major haplotypes A, B, C, and D from table 3, only the BD diplotype was associated with albuminuria (OR 3.03, 95% CI 1.36 - 6.79, p = 0.007, compared to individuals without the BD diplotype). However, this did not reach a Bonferroni corrected level of statistical significance of p < 0.005. The diplotypes consisting of the B or D "risk" haplotypes were rare, with BB, BD, and DD diplotypes accounting for 2.7%, 2.2%, and 0.5% of the 1663 European Americans with available diplotypes analyzed.

#### African Americans

Among African Americans, no *GLUT1 *SNP was significantly associated with case status. Frequency of the Promoter SNP genotype was slightly but not significantly different by case status (see figure 1). *Enh2 *TT genotype was rare (0.3% in controls, 0.0% in cases) and did not allow examination with case status. There was sufficient power to examine the XbaI SNP (with the minor A allele frequency of 42.4%) and case status in African Americans, which was not significant(P = 0.5). By diabetes status, the *XbaI *AA risk genotype was not associated with albuminuria either among those with type 2 diabetes (OR 1.05, 95% CI: 0.53 - 2.09) or without diabetes (OR 1.34, 95% CI: 0.70 - 2.56). Further detailed haplotype and diplotype analyses did not demonstrate any significant associations with albuminuria among African Americans (P = 0.877, table 3).

### *GLUT1 Enh2 *and Albuminuria in Samples from Stages 1 and 2

Based on stage 1, the *Enh2 *SNP appeared to be the risk polymorphism affecting albuminuria, particularly among European Americans with type 2 diabetes. The *GLUT1 Enh2 *polymorphism was genotyped in a separate stage 2 of European Americans (n = 6319; 374 cases) and African Americans (n = 1021, 146 cases), and the risk genotype tended towards an increased (but not statistically significant) OR among European Americans with diabetes (OR = 1.66, 95% CI: 0.77 - 3.57, p = 0.192) but not among non-diabetics (OR = 0.99, 95% CI: 0.61 - 1.59, p = 0.953). The risk association among European Americans with diabetes from stage 1 was not replicated in stage 2. In the stage 2 sample of African Americans, the GLUT1 *Enh2 *risk genotype was still rare (0.1% of controls and 1.4% of cases, Chi-square(2d.f.) P = 0.03) precluding informative analysis by diabetes status.

By race, we combined the samples from stages 1 and 2 for European Americans (n = 8,122) and African Americans (n = 2156) and examined risk-factor adjusted associations with albuminuria and the *GLUT1 *Enh2 risk genotype by diabetes status (Table 4). Among European Americans with diabetes, the *Enh2 *risk genotype had an increased risk-factor adjusted OR with albuminuria (OR 2.11, p = 0.010), microalbuminuria (OR 2.01, p = 0.034) and macroalbuminuria (OR 2.69, p = 0.045) (Model 1, Table 4). Further adjustment for BMI and estimated renal function (model 2) did not significantly affect the associations (with albuminuria OR 2.14, p = 0.009), nor did further adjustment for fasting glucose (OR 1.99, p = 0.029). However, among European Americans without type 2 diabetes, Enh2 was not associated with albuminuria (OR 1.01, p = 0.980) and was moderately but not significantly associated with macroalbuminuria (OR 1.64, 95% CI: 0.64-4.25, p = 0.305) (Model 1, Table 4). Additional adjustment for visit 2 hemoglobin A1C (HbA1C) in a sub-sample did not significantly change point estimates with albuminuria (OR = 1.87, 95% CI: 1.02 - 3.42 in diabetic individuals, OR = 1.25, 95% CI: 0.63 - 2.46 in non-diabetics). There was no evidence of interaction with hypertension (results not shown). Additionally, among European Americans, we also examined potential associations using multivariate linear regression with log-transformed ACR μg/mg (data log-transformed due to the skewed distribution) and also serum creatinine among European Americans, stratified by type 2 diabetes (see Table 5). Though the Enh2 risk genotype tended to be positively associated with worse ACR and higher serum creatinine only among type 2 diabetic European Americans, there were no statistically significant associations. We did not perform this analysis among African Americans given the low frequency of the Enh2 risk genotype among this population.

**Table 4:** Adjusted relative odds of albuminuria, microalbuminuria, and macroalbuminuria for *Enh 2 *in all genotyped European Americans and African Americans, by type 2 diabetes status.

	*GLUT1 Enh2 *Risk Genotype(TT) Relative Odds (95% Confidence Interval) reference: (CC or CT)					
**Model**^† ^**(N = Enh2 TT Genotype/All Genotypes)**	**ALBUMINURIA (N Cases = Cases with Enh2 TT Genotype/All Cases)**		**MICROALBUMINURIA (N Cases = Cases with Enh2 TT Genotype/All Cases)**		**MACROALBUMINURIA (N Cases = Cases with Enh2 TT Genotype/All Cases)**	

**EUROPEAN AMERICANS**	**OR (95% CI)**	**P-Value**	**OR (95% CI)**	**P-Value**	**OR (95% CI)**	**P-Value**

**TYPE 2 DIABETES (N = 72/1,095)**	**(N Cases = 20/187)**		**(N Cases = 14/134)**		**(N Cases = 6/53)**	
Model 1	2.11 (1.19 - 3.73) *	0.010	2.01 (1.06 - 3.82) *	0.034	2.69 (1.02 - 7.09) *	0.045
Model 2	2.14 (1.20 - 3.80) **	0.009	2.00 (1.05 - 3.81) *	0.035	2.66 (0.98 - 7.26)	0.056
Model 2 + Fasting Glucose^§^	1.99 (1.07 - 3.68) *	0.029	2.07 (1.06 - 4.06) *	0.034	1.85 (0.58 - 5.91)	0.299

**NON-DIABETIC (N = 478/7,027)**	**(N Cases = 24/344)**		**(N Cases = 19/298)**		**(N Cases = 5/46)**	

Model 1	1.01 (0.65 - 1.55)	0.980	0.91 (0.56 - 1.48)	0.713	1.64 (0.64 - 4.25)	0.305
Model 2	1.03 (0.67 - 1.59)	0.899	0.93 (0.57 - 1.50)	0.764	1.77 (0.67 - 4.64)	0.246
Model 2 + Fasting Glucose^§^	1.05 (0.68 - 1.63)	0.815	0.95 (0.59 - 1.54)	0.843	1.78 (0.68 - 4.66)	0.242

**AFRICAN AMERCIANS**	**OR (95% CI)**	**P-Value**	**OR (95% CI)**	**P-Value**		

**TYPE 2 DIABETES (N = 2/573)**	**(N Cases = 1/160)**		**(N Cases = 1/107)**		**(N Cases = 0/53)**	

Model 1	3.09 (0.18 - 54.14)	0.440	5.37 (0.32 - 90.91)	0.244		
Model 2	4.36 (0.24 - 79.81)	0.321	6.39 (0.37 - 110.75)	0.203		
Model 2 + Fasting Glucose^§^	7.26 (0.38 - 137.93)	0.187	8.12 (0.44 - 148.09)	0.158		

**NON-DIABETIC (N = 4/1583)**	**(N Cases = 1/134)**		**(N Cases = 1/113)**		**(N Cases = 0/21)**	

Model 1	3.29 (0.33 - 32.84)	0.310	4.14 (0.41 - 41.44)	0.227		
Model 2	4.69 (0.47 - 46.58)	0.187	5.15 (0.51 - 51.66)	0.164		
Model 2 + Fasting Glucose	8.07 (0.70 - 93.63)	0.095	8.60 (0.73 - 100.97)	0.087		

Statistical significance of point estimates: * P < .05 | ** P < .01 | *** P < .001

^† ^Analyses with microalbuminuria as the outcome excluded macroalbuminuria cases. Analyses with macroalbuminuria as the outcome excluded microalbuminuria cases. Among African Americans, analyses with macroalbuminuria were omitted due to extremely low allele frequency.

Model 1 includes age, sex, systolic blood pressure, diastolic blood pressure and hypertension medication use.

Model 2 includes Model 1 covariates and BMI and estimated glomerular filtration rate.

^§ ^Model 2 + Fasting Glucose is limited to individuals with fasting glucose values: non-diabetic European Americans N = 6,927 (340 albuminuria cases) and European Americans with type 2 diabetes N = 983 (166 albuminuria cases) and non-diabetic blacks N = 1,520 (126 albuminuria cases) and blacks with type 2 diabetes N = 415 (100 albuminuria cases).

Among European Americans, P-values estimating interaction between GLUT Enh2 and diabetes were not significant for the outcomes of albuminuria (P-interaction = 0.064), microalbuminuria (P-interaction = 0.071), and macroalbuminuria (P-interaction = 0.730). Among blacks, P-values estimating interaction between GLUT Enh2 and diabetes were not significant for the outcomes of albuminuria (P-interaction = 0.86), microalbuminuria (P-interaction = 0.96).

**Table 5:** Unadjusted and multivariate linear regression of ln (ACR) and serum creatinine for *Enh 2 *in all genotyped European Americans, by type 2 diabetes status.

	Regression coefficient for*GLUT1 Enh2 *Risk Genotype(TT) (95% CI) reference: (CC or CT)			
**(N = Enh2 TT Genotype/All Genotypes)**	**ln (ACR) ß Coefficient (95% CI)**	**P-value**	**Serum Creatinine ß Coefficient (95% CI)**	**P-value**

**TYPE 2 DIABETES (N = 72/1,095)**				

Unadjusted	0.26 (-0.17 - 0.69)	0.240	0.06 (-0.00 - 0.13)	0.069
Model 1	0.27 (-0.14 - 0.68)	0.196	0.02 (-0.04 - 0.09)	0.446
Model 2	0.28 (-0.13 - 0.68)	0.185	0.02 (-0.04 - 0.09)	0.446

**NON-DIABETIC (N = 478/7,027)**				
Unadjusted	-0.02 (-0.12 - 0.09)	0.737	-0.01 (-0.04 - 0.01)	0.189
Model 1	-0.02 (-0.12 - 0.08)	0.702	-0.01 (-0.03 - 0.01)	0.146
Model 2	-0.02 (-0.12 - 0.08)	0.667	-0.01 (-0.03 - 0.00)	0.143

Model 1 includes age, sex, systolic blood pressure, diastolic blood pressure and hypertension medication use.

Model 2 includes Model 1 covariates and BMI and estimated glomerular filtration rate for the outcome ln (ACR). For the outcome of serum creatinine, model 2 includes Model 1 covariates and BMI.

Among African Americans, after adjustment for model 2 covariates, the GLUT1 *Enh2 *risk genotype had a positive association with albuminuria and microalbuminuria among those with type 2 diabetes (OR = 4.36 and 6.39 with p = 0.321 and 0.203, respectively) and without diabetes (OR = 4.69 and 5.15 with p = 0.187 and 0.164, respectively), though it was not significant due to low frequency of the SNP.

### The *GLUT1 *Enh2 Risk Genotype and Insulin

**Figure 2:**
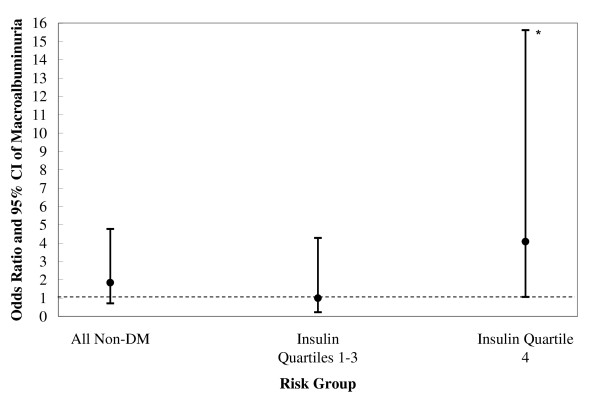
**Macroalbuminuria, *Enh2 *risk genotypes and insulin concentrations among non-diabetic European Americans (n = 6629 (46 cases))**. *P < 0.05. Among non-diabetic European Americans with fasting insulin, the risk of macroalbuminuria associated with the GLUT1 Enh2 TT risk genotype was 1.84, 95% CI: 0.71 - 4.77, P = 0.210, adjusting for age, gender, hypertension status, BMI, and GFR. For individuals in the upper quartile of insulin concentrations, the Enh2 TT genotype was associated with macroalbuminuria (OR 4.08, 95% CI: 1.06 - 15.61, p = 0.040) while it was not associated among those in the lower three quartiles (OR 1.00, 95% CI: 0.23 - 4.28, P = 0.995). There was no significant interaction between high concentrations of insulin and Enh2 TT genotypes on macroalbuminuria risk (P-interaction = 0.163).

To examine effects of insulin concentration on the association of GLUT1 *Enh2 *genotypes and albuminuria, we stratified by insulin concentrations excluding those with diabetes (to avoid confounding due to insulin treatment for diabetes). Due to the low frequency of the GLUT1 *Enh2 *risk genotype among African Americans, analysis was limited to European Americans. Among European Americans, (n = 6583 controls, 294 cases of microalbuminuria, and 46 cases of macroalbuminuria), the mean fasting insulin concentration was 10.9 μU/mL (S.D. = 7.5). Risk of albuminuria for *Enh2 *TT was OR 1.08 (95% CI: 0.70 - 1.67, p = 0.724), adjusting for age, gender, hypertension status, BMI, and GFR. Among individuals with insulin in the highest quartile (mean insulin 19.9 μU/mL, S.D. = 9.7), the adjusted OR of albuminuria for *Enh2 *TT was 1.25 (95% CI: 0.60 - 2.58, p = 0.547). Among individuals with insulin concentrations in the lower three quartiles (mean 7.9 μU/mL, S.D. = 2.9), the adjusted OR of albuminuria was 1.00 (95% CI: 0.58 - 1.72, p = 0.990) for *Enh2 *TT carriers. When we focused on the more specific outcome of macroalbuminuria as a phenotype and excluded patients with microalbuminuria, risk of macroalbuminuria for *Enh2 *TT was OR 1.84 (95% CI: 0.71 - 4.77, p = 0.210) as illustrated in figure 2. Among individuals with insulin in the highest quartile, the adjusted OR for *Enh2 *TT was 4.08 (95% CI: 1.06 - 15.61, p = 0.040). Among individuals with insulin concentrations in the lower three quartiles, the adjusted OR of macroalbuminuria was 1.00 (95% CI: 0.23 - 4.28; P = 0.995) for *Enh2 *TT carriers. Among non-diabetics, a formal test of interaction of the *GLUT1 Enh2 *risk genotype and the upper quartile of insulin was not significant for macroalbuminuria (P = 0.163).

## Discussion

Genetic variation of *GLUT1 *may be associated with the risk of micro- and macroalbuminuria in the general U.S. adult population of European Americans with type 2 diabetes. Though an association was seen when we combined European Americans with diabetes from stages 1 and 2, the findings of stage 1 were not replicated in stage 2. Both *XbaI *and *Enh2 *SNPs appeared to be associated in a recessive fashion with albuminuria, consistent with previous studies [5,6,8,11,13]. Further analysis showed Enh2 risk genotype (TT) increased risk independent of all other genotyped GLUT1 SNPs, including XbaI, suggesting that Enh2 is the causative GLUT1 SNP associated with albuminuria. Addtionally, no other GLUT1 SNPs besides *Enh2 *have been demonstrated to be located in a region with functional activity *in vitro*[5,6,8,13]. By study stage, the *Enh2 *risk genotype was associated with diabetic albuminuria among European Americans only in stage 1 and in the total study population; these findings were not replicated when we examined stage 2. In the total study population of European Americans with type 2 diabetes, the risk effect of Enh2 on macroalbuminuria was independent of age, gender, and hypertension. Possession of the Enh2 risk genotype and high insulin concentrations particularly increased macroalbuminuria risk among non-diabetic European Americans. Among African Americans, XbaI was not associated with albuminuria. The association of the *Enh2 *risk genotype and albuminuria among African Americans was increased among individuals with and without type 2 diabetes but was not statistically significant. Despite having over 2000 African Americans in the present study, the low frequency of the Enh2 risk genotype in this population (0.3%) limited the power of our analysis and prevents us from making definitive conclusions about the role of *GLUT1 *genetic variation and albuminuria in African Americans. However, our results suggest that GLUT1 Enh2 may be associated with albuminuria.

In European Americans, our results extend the findings of a prior study by Ng et al. among type 1 diabetics[35], suggesting both XbaI and Enh2 homozygotes as having an increased risk of albuminuria. However, the authors could not examine the independent risk effect of Enh2 or XbaI separately; since no individuals in their study possessed the Enh2 risk genotype but not the XbaI risk genotype. Multivariate analysis in our study suggest the XbaI AA risk genotype by itself does not confer risk (OR = 0.94, p = 0.931), whereas the Enh2 TT risk genotype (OR = 3.37, p = 0.090) has a stronger association with albuminuria independent of *XbaI*. Our results extend the risk association of Enh2 to albuminuria in European Americans with type 2 diabetes. Additionally, a recent study in Tunisia of type 2 diabetics had similarly demonstrated an association of the Enh2 TT risk genotype with nephropathy (OR 8.4 (95% CI: 3.3-21.5)) while finding no association with *Xba*I[11].

Additionally, our results suggest that among non-diabetic European Americans with the *Enh2 *risk genotype, those with higher concentrations of insulin were at greater risk of having proteinuria. The putative human enhancer-2 is homologous with the murine enhancer-2 which was responsive to insulin *in vitro*[5,24]. Furthermore, the Enh2 SNP is located within a binding site for the insulin-responsive transcription factor, the upstream stimulatory factor (USF) [5,25,26]. USF has been identified as a glucose-inducible transcription factor in mesangial cells [27,28] and shown to regulate GLUT1 transcription[29]. The Enh2 SNP of GLUT1 is located within a USF responsive element in humans, suggesting the TT genotype might have altered gene expression of GLUT1 that contributes to diabetic nephropathy[36]. Additionally, the interaction of the *Enh2 *SNP and insulin supports the previously posited hypothesis[5] that in individuals with the *Enh2 *risk genotype, high intracellular glucose concentrations might increase in mesangial cells in response to insulin[37]. The high concentrations of intracellular glucose may contribute to mesangial matrix expansion and glomerulosclerosis through several pathologic cellular mechanisms including the polyol pathway, activation of protein kinase C, increased formation of advanced glycation end-products, and the hexosamine pathway[38,39]. Additionally, there is evidence that podocytes are critical in maintaining the glomerular filtration barrier of the kidney and preventing albuminuria [40-42], and a recent study demonstrated that the glucose uptake of podocytes are insulin responsive and act via GLUT1, suggesting the insulin sensitivity of human podocytes resulting in urinary protein loss may act via these mechanisms [43]. Future studies should examine if the *Enh2 *SNP indeed modulates GLUT1 protein expression. Furthermore, most candidate gene chips (Affymetrix 5.0, 6.0, 500K, 100K; Illumina 550K, 650K) do not include *Enh2 *SNP. This suggests follow-up studies may need de-novo genotyping.

Previous studies of *XbaI *and diabetic nephropathy have had disparate results[12]. Our results for the *XbaI *risk genotype in European Americans are consistent with previous studies which demonstrated increased risk of type 1 diabetic nephropathy among those homozygous for the *XbaI *A allele[5,6], suggesting recessive transmission of the phenotype. Inconsistent results in other studies of *XbaI *may be due to different patterns of disequilibrium with the *Enh2 *polymorphism [7-11].

Our study has several limitations. Its design is cross-sectional and the albuminuria classification is based on a spot urine ACR because urine which was only collected at one ARIC visit. A phenotype based on a single measurement of ACR could definitely lead to incorrect conclusions, particularly with potential misclassification of borderline values with a dichotomous outcome such as albuminuria. However, sensitivity analyses for macroalbuminuria demonstrated a significant risk association with the *Enh2 *risk genotype. Another limitation is that the Enh2 risk genotype was only significantly associated with diabetic albuminuria among European Americans from stage 1 and in the combined analysis. The findings of stage 1 were not replicated in stage 2. The samples from stage 1 and 2 were not randomly sampled, with stage 1 consisting of a convenience sample enriched for those with type 2 diabetes and African Americans, a sample previously used for the study of genetic risk factors in cardiovascular disease and type 2 diabetes. Participants comprising stage 2 consisted of the remaining ARIC visit 4 particpants not in sample 1. However, *Enh2 *TT still had an increased (but not statistically significant) association with albuminuria among European Americans with diabetes from stage 2, and had a significant and strong association with diabetic albuminuria when we combined both stages. Additionally, despite the fact that our study included 8122 European Americans, of whom there were 1095 type 2 diabetics, given the rarity of the Enh2 TT risk genotype among diabetics (6.6%), the size of our study precludes any definitive conclusions regarding the association of type 2 diabetic albuminuria and the Enh2 polymorphism of *GLUT1*. Given this limitation, our study can at best suggest an association between Enh2 and albuminuria among European American type 2 diabetics; however, due to the limitation in our number of subjects, we do not have sufficient power to make conclusive statements regarding this association. Perhaps a larger study of type 2 diabetic European Americans involving multiple cohorts may provide more power to definitively evaluate the role of the Enh2 risk genotype of GLUT1 and albuminuria. Furthermore, we did not have hemoglobin A1C (HbA1C) for all participants in our sample, though in our sub-sample, adjustment did not significantly affect results. Instead, we used fasting glucose as a marker of glycemic control. However, Ng et al. had previously found the strength of the association of the *Enh2 *risk genotype and diabetic albuminuria was independent of both HbA1C and duration of diabetes[5]. Ours is the largest study of *GLUT1 *genetic variation and albuminuria to date and the first to examine associations among non-diabetics and African Americans. We substantiate prievous findings and extend the association beyond type 1 diabetes, demonstrating a role for *GLUT1 Enh2 *and proteinuria among those with type 2 diabetes.

## Conclusions

In summary, *GLUT1 *genetic variation of *Enh2 *may predict risk of micro- and macroalbuminuria among European Americans with type 2 diabetes. Though the *Enh2 *risk genotype was significantly associated with diabetic albuminuria among European Americans from stage 1 and in the combined analysis, the findings of stage 1 were not replicated in stage 2. Furthermore, the rarity of the *Enh2 *risk genotype among African Americans precludes any definitive conclusions, although data suggest a risk-enhancing role. Our results suggest the *Enh2 *SNP, and not *Xba*I, is the causative polymorphism associated with diabetic albuminuria. Additionally, the *Enh2 *risk genotype may interact with hyperinsulinemia to further increase susceptibility to albuminuria, consistent with hypotheses generated by laboratory data. Studying mechanisms mediating this association may shed light on novel pathways and therapeutic targets in the pathophysiology of albuminuria and nephropathy. The modest size of the risk associated with *GLUT1 Enh2 *variation limits utility for screening, risk stratification and individualized therapy. However, if multiple genes of small and moderate effect on nephropathy are identified, they may compose panels for risk assessment.

## Competing interests

The authors declare that they have no competing interests.

## Authors' contributions

CCH, WHLK, CWH, and JC designed the study. Laboratory work was undertaken by MWS, WHLK, TG, FLB, EB, and AS. Statistical analysis was done by CCH, WHLK, and JC. CCH wrote the first draft of the paper. All authors contributed to and approved of the final version of the manuscript.

## Pre-publication history

The pre-publication history for this paper can be accessed here:

http://www.biomedcentral.com/1471-2350/12/16/prepub
